# ATP-binding cassette transporters at the zebrafish blood-brain barrier and the potential utility of the zebrafish as an *in vivo *model

**DOI:** 10.20517/cdr.2021.35

**Published:** 2021-05-25

**Authors:** Jordan M. Hotz, Joanna R. Thomas, Emily N. Katz, Robert W. Robey, Sachi Horibata, Michael M. Gottesman

**Affiliations:** ^1^Laboratory of Cell Biology, Center for Cancer Research, National Cancer Institute, National Institutes of Health, Bethesda, MD 20892, USA.; ^2^Zebrafish Core, Eunice Kennedy Shriver National Institute of Child Health and Human Development, Bethesda, MD 20892, USA.

**Keywords:** ABC transporters, blood-brain barrier, ABCG2, P-glycoprotein, zebrafish

## Abstract

The brain is protected from toxins by a tightly regulated network of specialized cells, including endothelial cells, pericytes, astrocyes, and neurons, known collectively as the blood-brain barrier (BBB). This selectively permeable barrier permits only the most crucial molecules essential for brain function to enter and employs a number of different mechanisms to prevent the entry of potentially harmful toxins and pathogens. In addition to a physical barrier comprised of endothelial cells that form tight junctions to restrict paracellular transport, there is an active protective mechanism made up of energy-dependent transporters that efflux compounds back into the bloodstream. Two of these ATP-binding cassette (ABC) transporters are highly expressed at the BBB: P-glycoprotein (P-gp, encoded by the *ABCB1* gene) and ABCG2 (encoded by the *ABCG2* gene). Although a number of *in vitro* and *in vivo* systems have been developed to examine the role that ABC transporters play in keeping compounds out of the brain, all have inherent advantages and disadvantages. Zebrafish (*Danio rerio*) have become a model of interest for studies of the BBB due to the similarities between the zebrafish and mammalian BBB systems. In this review, we discuss what is known about ABC transporters in zebrafish and what information is still needed before the zebrafish can be recommended as a model to elucidate the role of ABC transporters at the BBB.

## INTRODUCTION

### The blood-brain barrier

**Figure 1:**
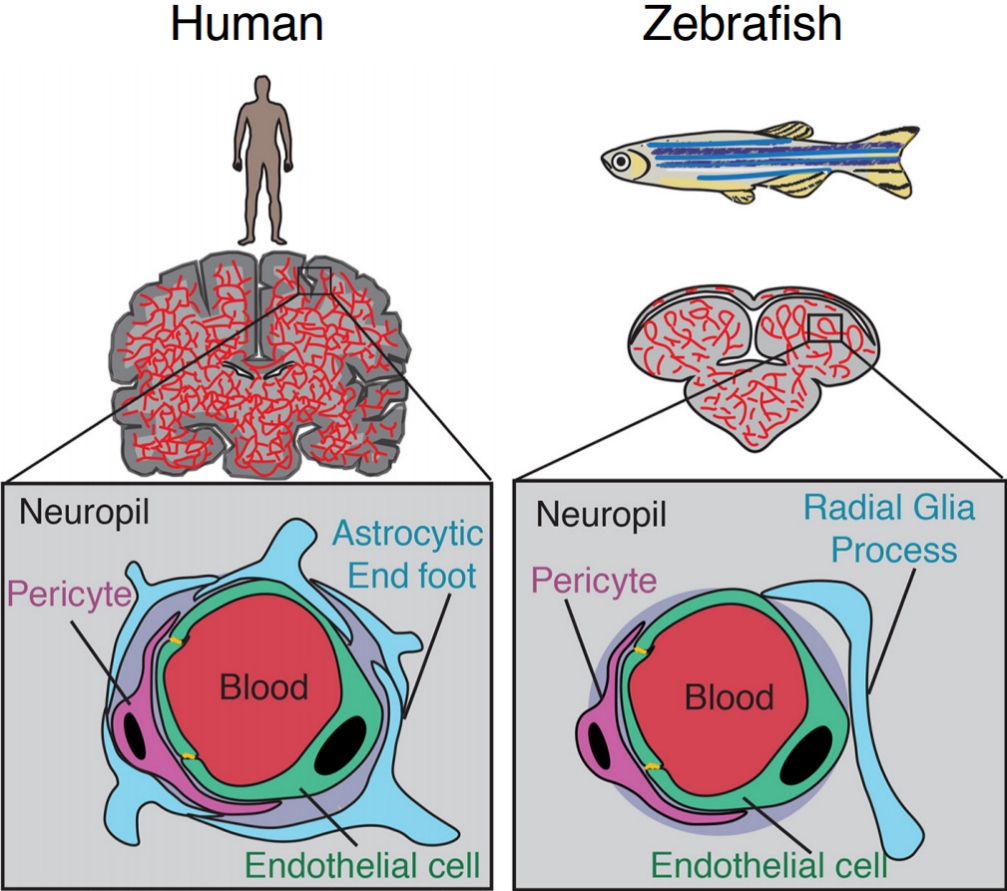
Comparison of the human and zebrafish neurovascular unit. Adapted from O’Brown *et al.*^[41]^.

The blood-brain barrier (BBB) restricts the free exchange of molecules between the blood and the brain and is required to maintain and protect the neural microenvironment. The BBB refers to the specialized adaptations of the microvasculature of the brain. The existence of the BBB was first reported when tracer dyes injected into the circulatory system were not observed to stain the brain^[1]^ and confirmed by the difference in lethality of poisons injected subcutaneously or into the central nervous system (CNS)^[2]^. The cerebral vascular endothelium, along with neurons, pericytes, astrocytes, and the extracellular matrix comprise the basic structural unit of the BBB, termed the neurovascular unit (NVU)^[3]^ [Figure 1]. The intimate physical and chemical links between the cells of the NVU allow for modulation of the BBB in response to stimuli such as inflammation, infection, injury, and neuronal energy requirements^[4-6]^. Other important components of the brain microenvironment include microglia, macrophages, and fibroblasts^[7]^.

The integrity of the BBB is attributable to key properties of the CNS vascular endothelial cells. Endothelial cells at the mammalian BBB are linked by tight junctions close to their apical membranes that limit paracellular diffusion. Tight junction transmembrane proteins contributing to the BBB are claudin-1, -3, -5, -12; occludin, and lipolysis-stimulated protein (LSR)^[8]^. Claudin-5 is the most abundant tight junction protein at the BBB^[9]^. Knockout of claudin-5 or LSR in mice leads to increased BBB permeability to small molecular weight compounds^[10,11]^. Occludin is believed to have a regulatory role in tight junctions and is not required for their formation^[12]^. The zonula occludens (ZO) family are intracellular scaffold proteins that link most tight junction transmembrane proteins to the actin cytoskeleton, stabilizing these cell-cell adhesions^[8]^. ZO-1 and ZO-2 are expressed in endothelial cells of the BBB, whereas ZO-3 is not^[13]^. ZO-1 and ZO-2 have functional redundancy in epithelial cells, and deletion of both results in a complete lack of tight junctions^[14]^. In human-derived microvascular endothelial cells, however, ZO-1 is required for tight junction formation and structural integrity, independently of ZO-2^[15]^. Endothelial cells at the BBB also have low levels of micropinocytosis in comparison to peripheral endothelial cells, limiting transcytosis^[16,17]^.

The remaining cell types that form the canonical NVU are pericytes and astrocytes. Pericytes are mural cells that partially cover the ablumenal endothelial walls of microvessels and are embedded in the vascular basement membrane. They form cell-cell contacts with endothelial cells and astrocytic end-feet^[18]^. Pericytes are required for the formation of the BBB^[19]^ and for maintenance of BBB function in adulthood^[20]^. Astrocytes, a type of glial cells that make up a significant portion of the CNS, form contacts with both microvessel walls and neurons, providing a cellular link between neurons and vasculature^[21]^. However, astrocytes are not required in the formation of the BBB, as a functional BBB is present in rodent embryogenesis prior to astrocyte differentiation^[19]^. Astrocytes are important in the modulation and maintenance of the BBB, and are able to induce the formation of barrier-like properties in non-CNS-derived endothelial cells, as demonstrated by transplant studies^[22]^ and co-culture experiments^[21,23]^.

CNS endothelial cells express two types of transporters: nutrient transporters and efflux transporters^[18]^. Nutrient transporters allow the entry of key nutrients, such as glucose and insulin, into the brain^[24]^. The efflux transporters expressed belong to the adenosine triphosphate (ATP) binding cassette (ABC) transporter family, which are encoded by a diverse family of 48 human genes. Some of these transporters have specific physiological substrates, whereas others can transport a broad range of exogenous compounds^[25]^. ABC transporters form a selective, active barrier protecting the brain from toxins, xenobiotics, and a wide range of drugs^[26]^. Two of the most highly expressed ABC transporters at the BBB are P-glycoprotein (P-gp, encoded by the *ABCB1* gene) and ABCG2 (encoded by the *ABCG2* gene)^[27]^. Throughout this review, we use the terms P-gp and ABCG2 to refer to the human proteins. ABCC family members such as ABCC2, ABCC4 and ABCC5 are also expressed on the lumenal surface of brain endothelial cells^[26]^. The significant role of P-gp at the BBB was demonstrated in 1994 by Schinkel *et al.*^[28]^, who found that deletion of the *Abcb1a* gene in mice caused a lethal 90-fold increase in brain penetration of the drug ivermectin that is used as an anti-parasitic. Deletion of the *Abcg2* gene in mice led to increased phototoxicity due to high blood levels of a chlorophyll catabolite^[29]^. Mice deficient in both murine homologs of human *ABCB1*, *Abcb1a* and *Abcb1b*, as well as the murine *Abcg2* gene have shown that both proteins work cooperatively to prevent entry of many chemotherapeutic agents into the brain^[25]^. Toxicity from ivermectin, used commonly to prevent and treat heartworm in dogs, occurs in American collies due to loss of expression of canine P-gp^[30]^ and humans lacking functional P-gp cannot tolerate ivermectin^[31]^. The multidrug efflux transporters, P-gp and ABCG2, both have wide substrate specificities and are therefore well-suited to protect the CNS from a wide range of toxins^[26]^.

The BBB is highly efficient at protecting the brain, with the impermeability and selectivity of the barrier resulting in exclusion of all large-molecule neurotherapeutics, and 98% of small molecule drugs^[32]^. The delivery of small molecule drugs into the brain for treatment of CNS-related diseases and brain cancers represents a significant challenge in drug development. ABC transporter expression at the BBB plays a pivotal role in preventing the brain penetration by these therapeutics. As a better understanding of the role of ABC transporters at the BBB could lead to the development of CNS-targeted therapeutics, several *in vitro* and *in vivo* models have been developed to facilitate research in this area.

### Models for studying ABC transporters at the BBB

*In vitro* and *in vivo* model systems have been used to explore the contributions of ABC transporters to the integrity of the BBB. Some of the more complex* in vitro* models permit visualization of the structure and function of the NVU cells, while *in vivo* models reveal gene expression patterns and membrane permeability through real-time imaging^[33,34]^. Identifying optimal models would aid in the development of effective therapeutic agents that can bypass the BBB and target specific neurological disease sites.

#### Transwell assays

Endothelial cell lines that form polarized monolayers and tight junctions were some of the first models used to examine the role transporters play in keeping substrates out of the brain. Transwell assays using Madin-Darby canine kidney (MDCK) or pig kidney (LLC-PK1) cells transfected with P-gp have routinely been used to identify substrates of P-gp. MDCK and LLC-PK1 cells have also been transfected to express both P-gp and ABCG2 to explore the effects of their coexpression^[35-37]^. Caco-2 colorectal adenocarcinoma cells also form tight junctions and express relatively high endogenous levels of P-gp and ABCG2. However, transwell assays are over-simplistic models that do not address the potential contributions of other cell types or blood flow in determining BBB permeability. Immortalized brain endothelial cell lines of human origin, such as the hCMEC/D3 line, or those of non-human origin, such as the murine cell lines MBEC4 and bEND.3, have also been used in transwell assays. However, these models have poor barrier properties, resulting in low levels of transendothelial electrical resistance and a poor BBB phenotype with weaker tight junction formation^[38]^. Additionally, transporter expression is much lower in the immortalized lines compared to primary cells.

#### Co-culture models

Neurovascular cell co-cultures are more advantageous models than monocultures of isolated brain endothelial cells because brain endothelial cells lose their BBB properties when they are removed from the brain microenvironment^[33]^. Combining endothelial cells with pericytes and astrocytes in a culture system stimulates the re-creation of the NVU environmental conditions for more accurate experimentation^[39]^. Such co-cultures simulate the BBB environment by inducing endothelial cell differentiation as well as by including BBB specific-tight junction proteins and ABC transporters^[33,34,40]^. Further developments have led to BBB-on-a-chip models, *in vitro* models that produce sheer stress for *in vivo *- like results concerning junction tightness and biomarker permeability^[39]^. Although the BBB-on-a-chip has overcome some limitations of the traditional transwell culture model, it is a flexible technology that can cause different forms of on-chip models to vary in microchannel size, cell type, and permeability measurement, which can lead to discrepancies in experimental results^[39]^.

#### Mouse models

The mouse is the most common species used for modeling the human BBB due to its similar vasculature and cell composition^[34,41]^. Knockout mouse models have been instrumental in demonstrating the protective role that transporters play at the BBB. As mentioned previously, mice that lack both murine P-gp homologs (Abcb1a and Abcb1b) as well as murine Abcg2 have been used to show that many chemotherapeutic agents do not enter the brain due to the expression of these transporters^[25]^. In one example, brain penetration of the anaplastic lymphoma kinase inhibitor, brigatinib, was approximately 42-fold higher in mice lacking Abcb1a/Abcb1b/Abcg2 compared to wild-type mice^[42]^. While mice are frequently used as models to study the BBB, they are expensive and not amenable to high throughput analysis^[34,43]^.

Even with the improvement over time of both *in vitro* and *in vivo* models that mimic the BBB environment and physiology, there are still certain disadvantages of using these models. Overcoming the disadvantages associated with the current BBB models used to study the role of ABC transporters may advance drug development and treatment of neurological diseases. The zebrafish has several advantages over other *in vivo* models of the BBB and has been suggested as a model for examining the role of ABC transporters expressed at the brain endothelium.

## THE ZEBRAFISH AS A MODEL TO STUDY ABC TRANSPORTERS AT THE BBB

Zebrafish possess many characteristics that make them a suitable model for studying the BBB. They are small, tropical, fresh-water teleost fish growing only to about 3 cm in length as adults, and can be housed in fairly small aquaria. Zebrafish require much less maintenance than mice. After external egg fertilization, their embryos are transparent and develop rapidly, making them ideal for observing biological processes *in vivo*. Large numbers of embryos are produced, providing the opportunity for large-scale high-throughput studies. Small-molecule screens have identified compounds that inhibit biological processes, demonstrating the usefulness of zebrafish in the drug discovery process for human disease^[44]^. Embryonic zebrafish can be imaged easily on a wide range of microscopes, with potential for sub-cellular and super-resolution, especially when combined with post-fixation processing techniques such as a tissue clearance^[45]^ and expansion microscopy^[46]^. Many useful fluorescent transgenic lines have been created with cell-specific promoters, such as the glut1b:mCherry line, with specifically labeled brain endothelial cells^[47]^.

### Similarities between the zebrafish and human BBB

Zebrafish have a BBB that is structurally and functionally similar to that of mammals. Permeability studies with dyes and tracers, such as FITC-dextran and HRP, have demonstrated the presence of a functional BBB in both developing and adult zebrafish^[48-50]^. The acquisition of BBB properties occurs in brain endothelial cells during angiogenesis starting at 30 h post-fertilization (hpf)^[47,51]^, and the BBB continues to mature, with increasing restriction of lower molecular weight substances with age, assessed up to 10 days post-fertilization (dpf)^[49]^.

The basic structure of the NVU is similar to that of humans and mice, with the notable exception of a direct equivalent to astrocytes [Figure 1]^[41]^. Zebrafish brain endothelial cells have many of the same adaptations as their mammalian counterparts. Teleost fish have homologs for the majority of the claudin family of transmembrane tight junction proteins, including claudin-5^[52]^. Zebrafish also have homologs from the ZO protein family, such as ZO-1 [tight junction protein (*tjp*)*1a* and *tjp1b*], ZO-2 (*tjp2a* and *tjp2b*), and ZO-3 (*tjp3*). *Tjp1b* is the dominant gene expressed in the zebrafish brain^[53]^, suggesting commonality with mammalian BBB tight junctions for which ZO-1 is crucial^[15]^. Zebrafish express homologs of claudin-5 and ZO-1, which are essential to the integrity of the mammalian BBB in brain microvessels, where they are localized to endothelial cell-cell junctions^[48,51]^. Physical evidence of tight junctions between endothelial cells of the BBB has been observed at 3 dpf^[54]^ and 10 dpf by electron microscopy^[49]^.

Reduced transcytosis across endothelial cells is another important adaptation of the mammalian BBB. During early zebrafish development from 3-10 dpf, suppression of endothelial cell transcytosis correlates with a decrease in BBB permeability^[54]^. The mammalian transcytosis regulator protein, MFSD2A, functions to reduce transcytosis in murine CNS endothelial cells^[55]^, and has a conserved function in zebrafish. Zebrafish with morpholino knockdown or mutation of the zebrafish homolog, *mfsd2aa*, leads to increased BBB permeability^[54,56]^. Similar to humans, zebrafish CNS endothelial cells also express transporters that contribute to the functionality of the BBB, including nutrient transporters, such as Glut1^[47]^, and multidrug efflux transporters, which are discussed below.

In the mammalian BBB, pericytes are embedded in the vascular basement membrane in close proximity to endothelial cells, and are believed to play an important role in the formation and maintenance of the BBB^[18]^. Pericytes have the same localization in the zebrafish BBB, as evidence by electron microscopy at 3 dpf indicates^[54]^. Pericytes are associated with brain microvessels from 48 hpf, after which they continue to proliferate and migrate to cover the vascular endothelium until 5 dpf^[57,58]^. Zebrafish embryos with a missense mutation in *notch3* have a deficiency in pericyte cell numbers due to reduced proliferation of pericyte progenitors^[57]^. These mutant embryos have brain hemorrhages and a more permeable BBB, with an absence of endothelial or tight junction defects^[57]^, similar to mammals lacking pericytes^[20]^. A panel of mammalian pericyte marker genes were used in order to identify brain pericytes in zebrafish. Zebrafish pericytes expressed both *pdgfrb*, a *PDFRb* homolog, and *tagln2*, a smooth muscle α-actin homolog, but not *rgs5a*, *desmin a*, *desmin b*, or the *NG2 *homolog, *cspg4*^[57]^*.* Zebrafish pericytes appear to have both neural crest and mesoderm origin^[58]^, whereas mammalian pericytes develop only from the neural crest^[59]^. The exact role of zebrafish brain pericytes and the consequences of the differences between expression profiles and origins of zebrafish and mammalian pericytes remain unclear.

Zebrafish lack a direct equivalent of classic stellate mammalian astrocytes; however, Chen *et al.*^[60]^ recently identified a population of cells derived from radial glial cells that are remarkably similar to astrocytes and could potentially be considered an astrocytic equivalent. Through evolution, glia have diversified to include radial glia, astrocytes, microglia, oligodendrocytes, and ependymal cells; each with different functions in the brain^[61]^. In mammals, radial glial cells differentiate to give rise to both neurons and astrocytes^[62]^. Zebrafish radial glia and mature mammalian astrocytes share the expression of many proteins, such as glial fibrillary acidic protein (GFAP), glutamate aspartate transporter, and aquaporin-4, implying a molecular similarity^[60,63]^.

Astrocyte function depends on highly branched morphology, which allows contact with synapses, neurons, the vasculature, and other glial cells. Radial glial cells, in contrast, have a much simpler morphology, with fewer and longer processes than astrocytes^[61]^. The astrocyte-like population of radial glia, identified by Chen *et al.*^[60]^, have a complex branched morphology that is very similar to mammalian astrocytes. These radial glial cells expressing GFAP have processes that make contact with neurons, synapses^[60]^, and microvasculature^[48]^, demonstrating a similar spatial organization to mammalian astrocytes^[61]^. Functional conservation exists between mammalian astrocytes and zebrafish astrocyte-like radial glia. For example, both exhibit spontaneous calcium transients^[60,64]^ and respond to damage of the CNS^[65]^. Controversy remains in the field as to whether radial glial cells are a rudimentary equivalent of astrocytes in zebrafish, or if the identified population of more complex radial glial cells are in fact bona fide astrocytes. Regardless of the consensus, the function of these cells with regards to the BBB has yet to be determined.

### Zebrafish ABC transporters at the BBB

**Figure 2:**
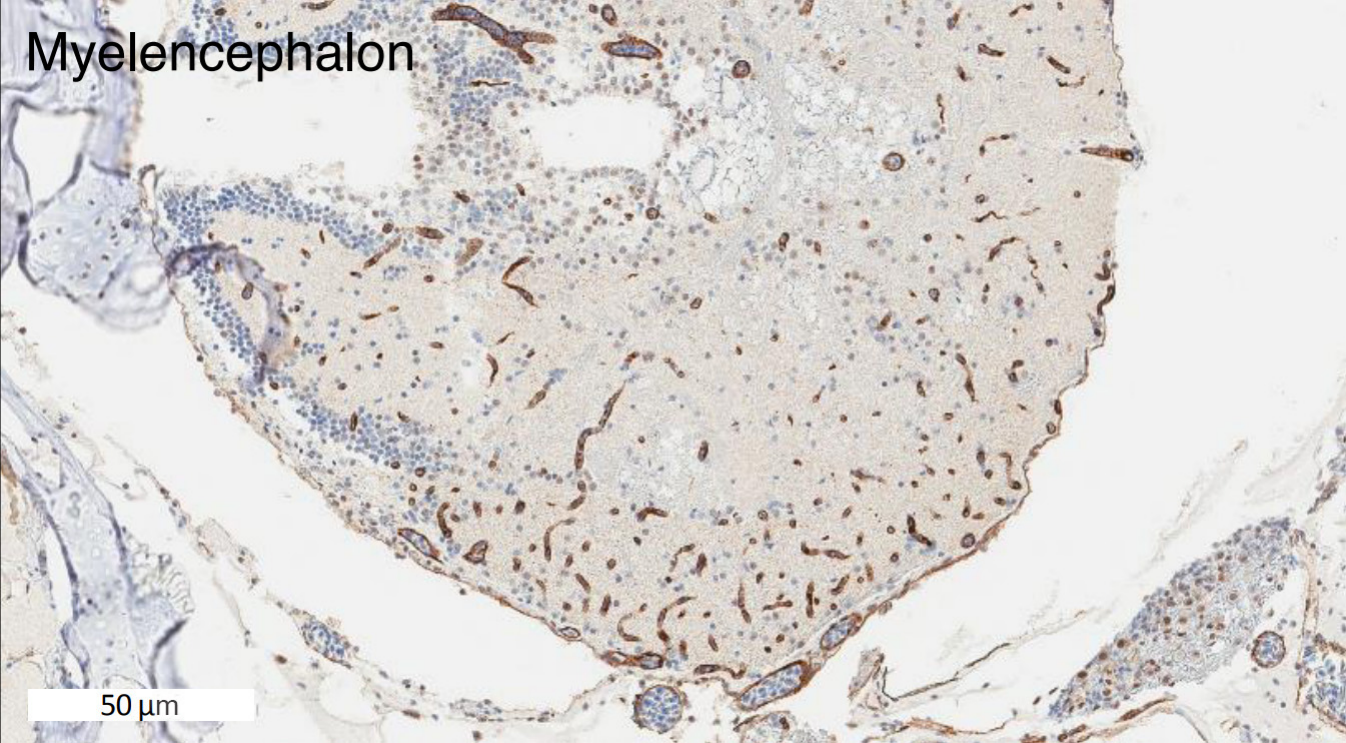
The C219 antibody reacts with cells in the zebrafish brain. Formalin-fixed, paraffin-embedded whole adult zebrafish were stained with the C219 antibody. Positive staining is observed in what appears to be the vasculature and is consistent with localization to the blood-brain-barrier.

Zebrafish have two homologs of human *ABCB1.* These two genes are currently named *abcb4 *and *abcb5*. They were previously designated as *abcb1b* and *abcb1a*, respectively, but were later renamed based on chromosomal localization^[66]^. The zebrafish Abcb4 protein has 63% amino acid similarity with human P-gp, while Abcb5 has 57% similarity^[49]^. Earlier studies showed that the C219 antibody, developed to detect human P-gp^[67]^, reacted with a protein found in zebrafish brain endothelial cells, likely due to preservation of the C219 epitope in zebrafish Abcb4 and Abcb5^[44,49]^. In our own studies, we have also noted C219 antibody reactivity in the vasculature of the zebrafish brain [Figure 2]. A transporter at the zebrafish BBB similar in function to P-gp has been demonstrated by functional assays with rhodamine 123, a known P-gp substrate. After injecting embryos at 3, 5, 8, and 10 dpf with rhodamine 123, Fleming *et al.*^[49] ^found that rhodamine was actively effluxed out of the brain at 8 dpf. In another study, rhodamine 123 was injected into the circulation of zebrafish embryos at 3, 4, 5, 6, and 7 dpf and brain fluorescence was monitored. At the earlier time points, dye was retained in the brain region, indicating a lack of transporter functionality. In the 6 and 7 dpf embryos, no fluorescence was observed, indicating that a homologous transporter was preventing brain penetration^[68]^. However, it is unclear whether one or both of the P-gp homologs are expressed at the zebrafish BBB and it is not known whether the substrate specificity of the transporters is comparable to that of P-gp. Known inhibitors of human P-gp have been used to demonstrate that a P-gp homolog contributes to the BBB integrity of other teleost fish, including killifish, rainbow trout, and dogfish sharks^[69-72]^.

For *ABCG2*, four homologs in the zebrafish have been identified: *abcg2a*, *abcg2b*, *abcg2c*, and *abcg2d*^[73]^. Comparison of the amino acid sequence of the zebrafish homologs with human ABCG2 revealed Abcg2a to have the highest amino acid sequence identity at 60%, followed by Abcg2d (48%), Abcg2c (45%), and Abcg2b (44%)^[74]^. Based on synteny analyses, *abcg2d* is the closest homolog of human *ABCG2*^[71]^. Although it is reasonable to assume that one, or possibly more, of the zebrafish ABCG2 homologs is or are expressed at the BBB, to date, no studies have localized any of them to the brain vasculature. Cells transfected to overexpress zebrafish Abcg2a were found to transport Hoechst 33342, but this appears to be the only known substrate of the zebrafish ABCG2 homologs^[74]^. Further studies are needed to confirm the existence of an ABCG2 homolog, or homologs at the zebrafish BBB and to determine the substrate specificity of the proteins.

Zebrafish also express homologs of human *ABCC2*, *ACBCC4* and *ABCC5*, which are localized to brain endothelial cell lumenal membranes in mammalian systems. Zebrafish express *ABCC4* and *ABCC5* in the brain, although localization by cell type has not been determined^[75,76]^. There is no evidence yet for expression of *ABCC2* in the zebrafish brain. However, it is expressed and is functional in isolated brain capillaries of killifish, which are also teleost fish^[72]^.

### Potential problems with the zebrafish model

Because of the noted similarities between the zebrafish and the mammalian BBB, the zebrafish has been suggested as a good model to study the role of transporters at the BBB^[43]^. In one comprehensive study, Kim *et al.*^[77] ^compared the zebrafish brain penetration of several compounds to penetration of the mouse brain. Among the compounds tested was loperamide, an analog of which has been explored for use as a PET imaging agent to measure P-gp function at the BBB^[78]^. The estimated partition coefficient for loperamide after oral administration in the zebrafish was found to increase by about 2-fold when coadministered with the P-gp inhibitor tariquidar, which was comparable to the 4-fold increase observed in mice. For several of the compounds tested, including both compounds known to cross the BBB and those that do not, the partition coefficients were similar in zebrafish and mice. However, for a subset of unknown compounds unique to the study, the partition coefficients for the zebrafish did not correlate with those of mice. As suggested by the authors, differences in influx or efflux transporters could be the reason for the discrepancy.

Indeed, this is one of the problems with suggesting that the zebrafish could be used to model transporter activity at the BBB. The assumption is that zebrafish transporters function much like human transporters. However, there is a paucity of data with regard to the substrate specificity of homologous zebrafish transporters. Fischer *et al.*^[66] ^examined the accumulation of P-gp substrates rhodamine B and calcein-AM in whole embryo assays and demonstrated increased fluorescence in the embryos treated with the substrates and P-gp inhibitors compared to the embryos treated with the substrates alone. In a subsequent study, a number of compounds were also found to increase rhodamine B fluorescence in embryos at 72 hpf, potentially due to inhibition of zebrafish Abcb4^[79]^. The cyanobacterial toxin microcystin-LR was also found to be a substrate of Abcb4 based on cytotoxicity assays with LLC-PK1 cells transfected with zebrafish *abcb4* as well as microinjection of zebrafish embryos with *abcb4*^[80]^. Expression of *Abcb5* was recently localized to zebrafish ionocytes and the fluorescent P-gp substrates calcein-AM and DiOC6 were transported, demonstrating that Abcb4 and Abcb5 can efflux known P-gp substrates. These few studies report what little is known about the substrate specificities of the zebrafish transporters that are homologous to P-gp.

**Figure 3:**
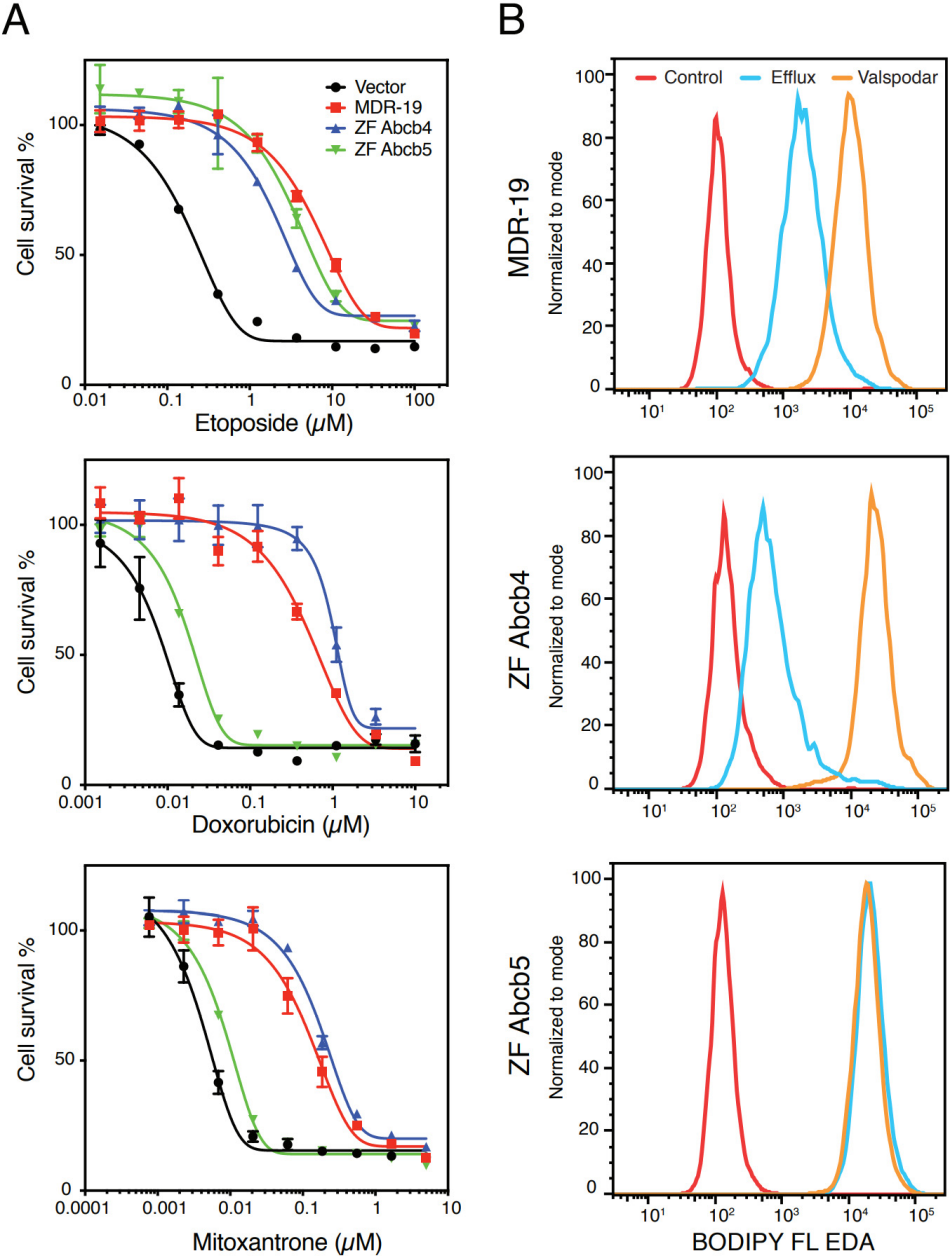
Comparison of the substrate specificity of human P-gp and zebrafish homologs. (A) Three-day cytotoxicity assays were performed with etoposide, doxorubicin or mitoxantrone on empty-vector-transfected HEK293 cells (Vector), HEK293 cells that express human P-gp (MDR-19), zebrafish Abcb4 (ZF Abcb4), or zebrafish Abcb5 (ZF Abcb5). (B) MDR-19, ZF Abcb4 and ZF Abcb5 cells were incubated with 500 nM BODIPY-ethylenediamine (EDA) in the presence or absence of 10 µM valspodar for 30 min. The medium was subsequently removed and replaced with substrate-free medium continuing without or with valspodar for another 1 h, after which intracellular fluorescence was measured on a flow cytometer.

To determine the substrate specificity of Abcb4 and Abcb5, we transfected HEK293 cells with empty vector (Vector) or with vectors encoding human *ABCB1* (MDR-19), zebrafish *abcb4* (ZF Abcb4), or zebrafish *abcb5* (ZF Abcb5). We then performed cytotoxicity assays with the known human P-gp substrates etoposide, doxorubicin, and mitoxantrone. As shown in Figure 3A, zebrafish Abcb4, zebrafish Abcb5, and P-gp conferred comparable levels of resistance to etoposide, while zebrafish Abcb5 conferred much less resistance to doxorubicin and mitoxantrone, suggesting that the substrate specificity profile of zebrafish Abcb5 might be narrower compared to zebrafish Abcb4 and P-gp. Further evidence was garnered from flow cytometry assays with fluorescent substrates [Figure 3B]. When we incubated MDR-19, ZF Abcb4, and ZF Abcb5 with the fluorescent substrate BODIPY-ethylenediamine (EDA) in the presence or absence of the P-gp inhibitor valspodar, we noted decreased intracellular fluorescence of BODIPY-EDA in the MDR-19 and ZF Abcb4 cells that was reversed by the addition of valspodar. However, transport was not observed in the ZF Abcb5 cells. Since these results pointed to a difference in substrate specificity between the zebrafish homologs, we performed a high-throughput screen on the transfected cells using 90 substrates of P-gp^[81]^. By comparing the drug response area under the curve for cells transfected with empty vector and the different transporters, we found that the substrate specificity profile of zebrafish Abcb4 is similar to that of human P-gp^[82]^. Additionally, we found that the efficacy of human P-gp inhibitors varied for zebrafish Abcb4 and Abcb5 and depended on the substrate examined. Using RNAScope *in situ* probes, we noted that zebrafish *abcb4* colocalized with C219 staining in the zebrafish brain vasculature. Vessels in the zebrafish brain that were claudin-5 positive also stained positively with *abcb4* probes^[82]^. These results support the hypothesis that zebrafish Abcb4 is the functional homolog of human P-gp at the zebrafish BBB.

**Figure 4:**
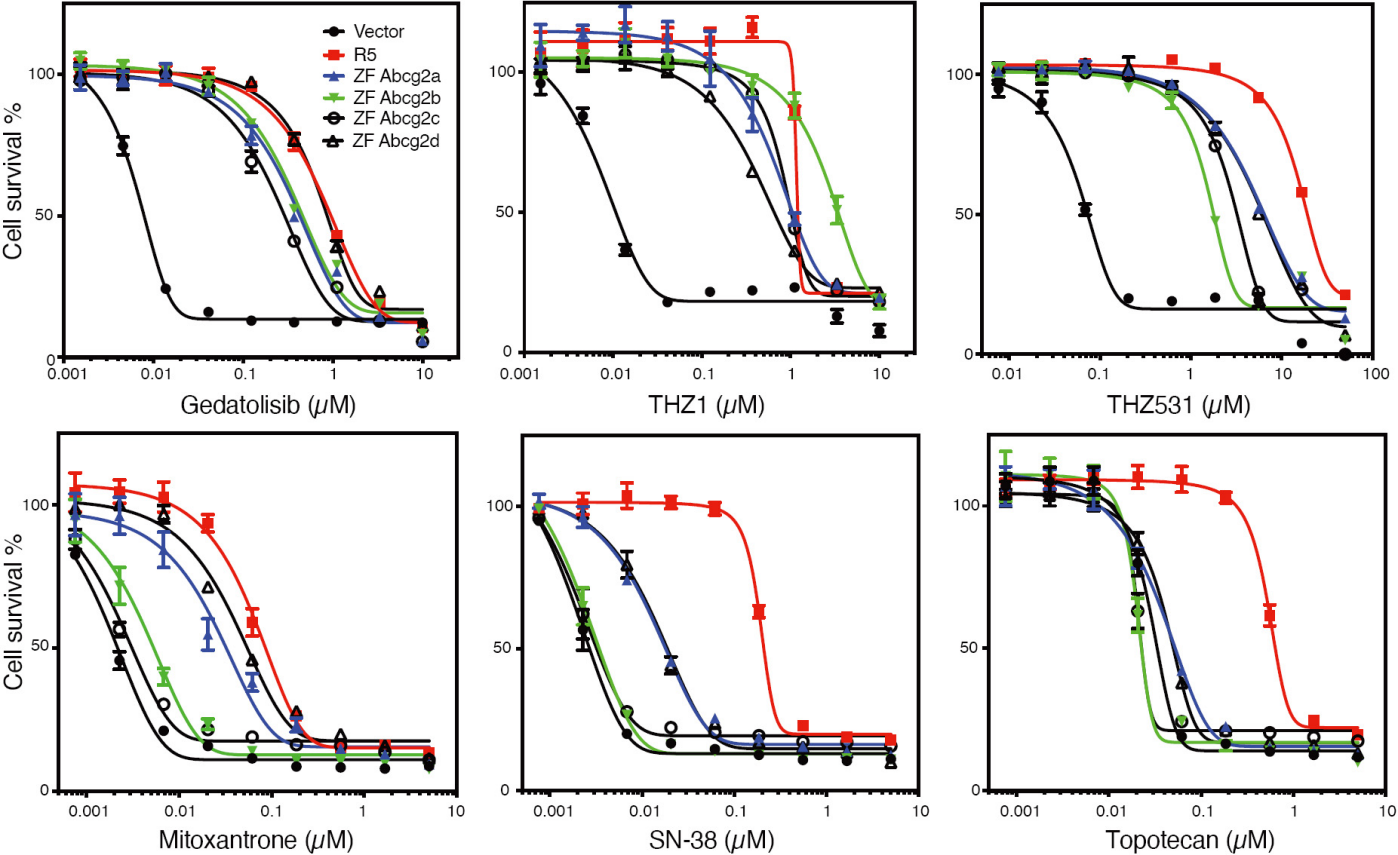
Zebrafish ABCG2 homologs do not functionally mimic human ABCG2. Three-day cytotoxicity assays were performed with gedatolisib, THZ1, THZ531, mitoxantrone, SN-38, or topotecan on empty-vector-transfected HEK293 cells (Vector), or HEK293 cells that express human *ABCG2* (R5), zebrafish *Abcg2a* (ZF Abcg2a), *Abcg2b* (ZF Abcg2b), *Abcg2c* (ZF Abcg2c), or zebrafish *Abcg2d* (ZF Abcg2d).

With regard to ABCG2, even less is known about transport substrates, with Hoechst 33342 being the only confirmed substrate for zebrafish Abcg2a^[74]^. In preliminary studies with HEK293 cells transfected with empty vector (Vector), or with vectors encoding human *ABCG2* (R5), zebrafish *abcg2a* (ZF Abcg2a), zebrafish *abcg2b* (ZF Abcg2b), zebrafish *abcg2c* (ZF Abcg2c), or zebrafish *abcg2d* (ZF Abcg2d), we noted very different substrate specificities in the zebrafish homologs as compared to human ABCG2. As shown in Figure 4, all of the zebrafish forms conferred similar levels of resistance to the known ABCG2 substrates THZ531, THZ1^[83]^, and gedatolisib^[81]^. However, stark differences were noted for mitoxantrone, SN-38 and topotecan, as only Abcg2a and Abcg2d conferred appreciable resistance to mitoxantrone or SN-38, and none of the zebrafish homologs conferred resistance to topotecan. Thus, none of the zebrafish homologs can be considered functionally equivalent to human ABCG2 based on our preliminary data, although further confirmation is necessary.

Further clarification is also needed with regard to when ABC transporters are expressed at zebrafish brain endothelial cells. Fleming *et al.*^[49]^ demonstrated colocalization of the C219 antibody within the vasculature of 8 dpf embryos, but not in those of 3 dpf embryos. Similarly, they noted exclusion of rhodamine 123 from the brain in 8 and 10 dpf embryos, but not in 5 dpf embryos. However, it is not yet clear if a zebrafish P-gp homolog is localized to the vasculature between 5 and 8 dpf. Additionally, no data are available with regard to ABCG2 homologs, so it is not clear if ABCG2 colocalizes at the same time as the P-gp homolog or if it follows a different timeline. Knowing the timepoint of BBB closure in zebrafish is important before proceeding with experimentation, especially for potential translational studies.

## CONCLUSIONS

Due to the highly conserved nature of zebrafish and mammalian BBB systems, as well as the small size and relatively ease of care of the zebrafish, it has emerged as a potentially useful model system for the study of ABC transporters at the BBB. Additionally, the ease of manipulating zebrafish genetics may lead to discoveries concerning maintenance of BBB integrity. Although zebrafish Abcb4 does appear to functionally copy P-gp at the zebrafish BBB, significant questions remain in terms of the substrate specificity of the ABCG2 homologs and their localization. If preliminary data gleaned from cell line models hold true, genetic modifications leading to humanization of the transporters could be possible; mice expressing human *ABCB1* have already been generated^[84]^. In any case, we anticipate that zebrafish will play a role in the development of therapeutic agents that can circumvent ABC transporters at the BBB, allowing improved brain penetration and more effective treatment.

## DECLARATIONS

### Copyright

The Author(s) 2021.

## References

[1] Ehrlich P

[2] Lewandowsky M (1900). Zur Lehre der Zerebrospinalflussigkeit. Z Klin Med.

[3] Hawkins BT, Davis TP (2005). The blood-brain barrier/neurovascular unit in health and disease. Pharmacol Rev.

[4] Blanchette M, Daneman R (2015). Formation and maintenance of the BBB. Mech Dev.

[5] Muoio V, Persson PB, Sendeski MM (2014). The neurovascular unit - concept review. Acta Physiol (Oxf).

[6] Neuwelt EA (2004). Mechanisms of disease: the blood-brain barrier. Neurosurgery.

[7] Zhao X, Chen R, Liu M, Feng J, Chen J, Hu K (2017). Remodeling the blood-brain barrier microenvironment by natural products for brain tumor therapy. Acta Pharm Sin B.

[8] Greene C, Hanley N, Campbell M (2019). Claudin-5: gatekeeper of neurological function. Fluids Barriers CNS.

[9] Ohtsuki S, Yamaguchi H, Katsukura Y, Asashima T, Terasaki T (2008). mRNA expression levels of tight junction protein genes in mouse brain capillary endothelial cells highly purified by magnetic cell sorting. J Neurochem.

[10] Sohet F, Lin C, Munji RN (2015). LSR/angulin-1 is a tricellular tight junction protein involved in blood-brain barrier formation. J Cell Biol.

[11] Nitta T, Hata M, Gotoh S (2003). Size-selective loosening of the blood-brain barrier in claudin-5-deficient mice. J Cell Biol.

[12] Saitou M, Furuse M, Sasaki H (2000). Complex phenotype of mice lacking occludin, a component of tight junction strands. Mol Biol Cell.

[13] Inoko A, Itoh M, Tamura A, Matsuda M, Furuse M, Tsukita S (2003). Expression and distribution of ZO-3, a tight junction MAGUK protein, in mouse tissues. Genes Cells.

[14] Umeda K, Ikenouchi J, Katahira-Tayama S (2006). ZO-1 and ZO-2 independently determine where claudins are polymerized in tight-junction strand formation. Cell.

[15] Tornavaca O, Chia M, Dufton N (2015). ZO-1 controls endothelial adherens junctions, cell-cell tension, angiogenesis, and barrier formation. J Cell Biol.

[16] Reese TS, Karnovsky MJ (1967). Fine structural localization of a blood-brain barrier to exogenous peroxidase. J Cell Biol.

[17] Chow BW, Gu C (2017). Gradual suppression of transcytosis governs functional blood-retinal barrier formation. Neuron.

[18] Daneman R, Prat A (2015). The blood-brain barrier. Cold Spring Harb Perspect Biol.

[19] Daneman R, Zhou L, Kebede AA, Barres BA (2010). Pericytes are required for blood-brain barrier integrity during embryogenesis. Nature.

[20] Armulik A, Genové G, Mäe M (2010). Pericytes regulate the blood-brain barrier. Nature.

[21] Abbott NJ, Rönnbäck L, Hansson E (2006). Astrocyte-endothelial interactions at the blood-brain barrier. Nat Rev Neurosci.

[22] Janzer RC, Raff MC (1987). Astrocytes induce blood-brain barrier properties in endothelial cells. Nature.

[23] Igarashi Y, Utsumi H, Chiba H (1999). Glial cell line-derived neurotrophic factor induces barrier function of endothelial cells forming the blood-brain barrier. Biochem Biophys Res Commun.

[24] Fu BM (2018). Transport across the blood-brain barrier. Adv Exp Med Biol.

[25] Robey RW, Pluchino KM, Hall MD, Fojo AT, Bates SE, Gottesman MM (2018). Revisiting the role of ABC transporters in multidrug-resistant cancer. Nat Rev Cancer.

[26] Hartz AM, Bauer B (2011). ABC transporters in the CNS - an inventory. Curr Pharm Biotechnol.

[27] Schinkel AH, Jonker JW (2003). Mammalian drug efflux transporters of the ATP binding cassette (ABC) family: an overview. Adv Drug Deliv Rev.

[28] Schinkel AH, Smit JJ, van Tellingen O (1994). Disruption of the mouse mdr1a P-glycoprotein gene leads to a deficiency in the blood-brain barrier and to increased sensitivity to drugs. Cell.

[29] Jonker JW, Buitelaar M, Wagenaar E (2002). The breast cancer resistance protein protects against a major chlorophyll-derived dietary phototoxin and protoporphyria. Proc Natl Acad Sci U S A.

[30] Mealey KL, Bentjen SA, Gay JM, Cantor GH (2001). Ivermectin sensitivity in collies is associated with a deletion mutation of the mdr1 gene. Pharmacogenetics.

[31] Baudou E, Lespine A, Durrieu G (2020). Serious ivermectin toxicity and human ABCB1 nonsense mutations. N Engl J Med.

[32] Pardridge WM (2005). The blood-brain barrier: bottleneck in brain drug development. NeuroRx.

[33] Canfield SG, Stebbins MJ, Morales BS (2017). An isogenic blood-brain barrier model comprising brain endothelial cells, astrocytes, and neurons derived from human induced pluripotent stem cells. J Neurochem.

[34] Jackson S, Meeks C, Vezina A, Robey RW, Tanner K, Gottesman MM (2019). Model systems for studying the blood-brain barrier: Applications and challenges. Biomaterials.

[35] Poller B, Wagenaar E, Tang SC, Schinkel AH (2011). Double-transduced MDCKII cells to study human P-glycoprotein (ABCB1) and breast cancer resistance protein (ABCG2) interplay in drug transport across the blood-brain barrier. Mol Pharm.

[36] Pastan I, Gottesman MM, Ueda K, Lovelace E, Rutherford AV, Willingham MC (1988). A retrovirus carrying an MDR1 cDNA confers multidrug resistance and polarized expression of P-glycoprtein in MDCK cells. ProcNatlAcadSciUSA.

[37] Fung KL, Kapoor K, Pixley JN (2016). Using the BacMam baculovirus system to study expression and function of recombinant efflux drug transporters in polarized epithelial cell monolayers. Drug Metab Dispos.

[38] Elbakary B, Badhan RKS (2020). A dynamic perfusion based blood-brain barrier model for cytotoxicity testing and drug permeation. Sci Rep.

[39] Bhalerao A, Sivandzade F, Archie SR, Chowdhury EA, Noorani B, Cucullo L (2020). In vitro modeling of the neurovascular unit: advances in the field. Fluids Barriers CNS.

[40] Helms HC, Abbott NJ, Burek M (2016). In vitro models of the blood-brain barrier: An overview of commonly used brain endothelial cell culture models and guidelines for their use. J Cereb Blood Flow Metab.

[41] O'Brown NM, Pfau SJ, Gu C (2018). Bridging barriers: a comparative look at the blood-brain barrier across organisms. Genes Dev.

[42] Li W, Sparidans RW, Wang Y, Lebre MC, Beijnen JH, Schinkel AH (2018). P-glycoprotein and breast cancer resistance protein restrict brigatinib brain accumulation and toxicity, and, alongside CYP3A, limit its oral availability. Pharmacol Res.

[43] Li Y, Chen T, Miao X (2017). Zebrafish: A promising in vivo model for assessing the delivery of natural products, fluorescence dyes and drugs across the blood-brain barrier. Pharmacol Res.

[44] Umans RA, Taylor MR (2012). Zebrafish as a model to study drug transporters at the blood-brain barrier. Clin Pharmacol Ther.

[45] Susaki EA, Tainaka K, Perrin D, Yukinaga H, Kuno A, Ueda HR (2015). Advanced CUBIC protocols for whole-brain and whole-body clearing and imaging. Nat Protoc.

[46] Wassie AT, Zhao Y, Boyden ES (2019). Expansion microscopy: principles and uses in biological research. Nat Methods.

[47] Umans RA, Henson HE, Mu F (2017). CNS angiogenesis and barriergenesis occur simultaneously. Dev Biol.

[48] Jeong JY, Kwon HB, Ahn JC (2008). Functional and developmental analysis of the blood-brain barrier in zebrafish. Brain Res Bull.

[49] Fleming A, Diekmann H, Goldsmith P (2013). Functional characterisation of the maturation of the blood-brain barrier in larval zebrafish. PLoS One.

[50] Quinonez-Silvero C, Hubner K, Herzog W (2020). Development of the brain vasculature and the blood-brain barrier in zebrafish. Dev Biol.

[51] van Leeuwen LM, Evans RJ, Jim KK (2018). A transgenic zebrafish model for the in vivo study of the blood and choroid plexus brain barriers using claudin 5. Biol Open.

[52] Loh YH, Christoffels A, Brenner S, Hunziker W, Venkatesh B (2004). Extensive expansion of the claudin gene family in the teleost fish, Fugu rubripes. Genome Res.

[53] Kiener TK, Sleptsova-Friedrich I, Hunziker W (2007). Identification, tissue distribution and developmental expression of tjp1/zo-1, tjp2/zo-2 and tjp3/zo-3 in the zebrafish, Danio rerio. Gene Expr Patterns.

[54] O'Brown NM, Megason SG, Gu C (2019). Suppression of transcytosis regulates zebrafish blood-brain barrier function. Elife.

[55] Ben-Zvi A, Lacoste B, Kur E (2014). Mfsd2a is critical for the formation and function of the blood-brain barrier. Nature.

[56] Guemez-Gamboa A, Nguyen LN, Yang H (2015). Inactivating mutations in MFSD2A, required for omega-3 fatty acid transport in brain, cause a lethal microcephaly syndrome. Nat Genet.

[57] Wang Y, Pan L, Moens CB, Appel B (2014). Notch3 establishes brain vascular integrity by regulating pericyte number. Development.

[58] Ando K, Fukuhara S, Izumi N (2016). Clarification of mural cell coverage of vascular endothelial cells by live imaging of zebrafish. Development.

[59] Korn J, Christ B, Kurz H (2002). Neuroectodermal origin of brain pericytes and vascular smooth muscle cells. J Comp Neurol.

[60] Chen J, Poskanzer KE, Freeman MR, Monk KR (2020). Live-imaging of astrocyte morphogenesis and function in zebrafish neural circuits. Nat Neurosci.

[61] Jurisch-Yaksi N, Yaksi E, Kizil C (2020). Radial glia in the zebrafish brain: functional, structural, and physiological comparison with the mammalian glia. Glia.

[62] Rowitch DH, Kriegstein AR (2010). Developmental genetics of vertebrate glial-cell specification. Nature.

[63] Grupp L, Wolburg H, Mack AF (2010). Astroglial structures in the zebrafish brain. J Comp Neurol.

[64] Di Castro MA, Chuquet J, Liaudet N (2011). Local Ca2+ detection and modulation of synaptic release by astrocytes. Nat Neurosci.

[65] Neve LD, Savage AA, Koke JR, Garcia DM (2012). Activating transcription factor 3 and reactive astrocytes following optic nerve injury in zebrafish. Comp Biochem Physiol C Toxicol Pharmacol.

[66] Fischer S, Kluver N, Burkhardt-Medicke K (2013). Abcb4 acts as multixenobiotic transporter and active barrier against chemical uptake in zebrafish (Danio rerio) embryos. BMC Biol.

[67] Georges E, Bradley G, Garipey J, Ling V (1990). Detection of P-glycoprotein isoforms by gene-specific monoclonal antibodies. Proc Natl Acad Sci USA.

[68] Park D, Haldi M, Seng W

[69] Bard SM, Gadbois S (2007). Assessing neuroprotective P-glycoprotein activity at the blood-brain barrier in killifish (Fundulus heteroclitus) using behavioural profiles. Mar Environ Res.

[70] Kennedy CJ, Tierney KB, Mittelstadt M (2014). Inhibition of P-glycoprotein in the blood-brain barrier alters avermectin neurotoxicity and swimming performance in rainbow trout. Aquat Toxicol.

[71] Luckenbach T, Fischer S, Sturm A (2014). Current advances on ABC drug transporters in fish. Comp Biochem Physiol C Toxicol Pharmacol.

[72] Miller DS, Graeff C, Droulle L, Fricker S, Fricker G (2002). Xenobiotic efflux pumps in isolated fish brain capillaries. Am J Physiol Regul Integr Comp Physiol.

[73] Annilo T, Chen ZQ, Shulenin S (2006). Evolution of the vertebrate ABC gene family: analysis of gene birth and death. Genomics.

[74] Kobayashi I, Saito K, Moritomo T, Araki K, Takizawa F, Nakanishi T (2008). Characterization and localization of side population (SP) cells in zebrafish kidney hematopoietic tissue. Blood.

[75] Long Y, Li Q, Li J, Cui Z (2011). Molecular analysis, developmental function and heavy metal-induced expression of ABCC5 in zebrafish. Comp Biochem Physiol B Biochem Mol Biol.

[76] Lu X, Long Y, Lin L, Sun R, Zhong S, Cui Z (2014). Characterization of zebrafish Abcc4 as an efflux transporter of organochlorine pesticides. PLoS One.

[77] Kim SS, Im SH, Yang JY (2017). Zebrafish as a screening model for testing the permeability of blood-brain barrier to small molecules. Zebrafish.

[78] Zoghbi SS, Liow JS, Yasuno F (2008). 11C-loperamide and its N-desmethyl radiometabolite are avid substrates for brain permeability-glycoprotein efflux. J Nucl Med.

[79] Bieczynski F, Burkhardt-Medicke K, Luquet CM, Scholz S, Luckenbach T (2020). Chemical effects on dye efflux activity in live zebrafish embryos and on zebrafish Abcb4 ATPase activity. FEBS Lett.

[80] Lu X, Long Y, Sun R, Zhou B, Lin L (2015). Zebrafish Abcb4 is a potential efflux transporter of microcystin-LR. Comp Biochem Physiol C Toxicol Pharmacol.

[81] Lee TD, Lee OW, Brimacombe KR (2019). A high-throughput screen of a library of therapeutics identifies cytotoxic substrates of P-glycoprotein. Mol Pharmacol.

[82] Robey RW, Robinson AN, Ali-Rahmani F (2021). Characterization and tissue localization of zebrafish homologs of the human ABCB1 multidrug transporter. bioRxiv.

[83] Gao Y, Zhang T, Terai H (2018). Overcoming resistance to the THZ series of covalent transcriptional CDK inhibitors. Cell Chem Biol.

[84] Yamasaki Y, Kobayashi K, Okuya F (2018). Characterization of P-glycoprotein humanized mice generated by chromosome engineering technology: its utility for prediction of drug distribution to the brain in humans. Drug Metab Dispos.

